# Size-tunable Lateral Confinement in Monolayer Semiconductors

**DOI:** 10.1038/s41598-017-03594-z

**Published:** 2017-06-12

**Authors:** Guohua Wei, David A. Czaplewski, Erik J. Lenferink, Teodor K. Stanev, Il Woong Jung, Nathaniel P. Stern

**Affiliations:** 1 Applied Physics Program, Northwestern University, 2145 Sheridan Road, Evanston, IL 60208 USA; 2 Center for Nanoscale Materials, Argonne National Laboratory, 9700 S Cass Avenue, Argonne, IL 60439 USA; 3 Department of Physics and Astronomy, Northwestern University, 2145 Sheridan Road, Evanston, IL 60208 USA

## Abstract

Three-dimensional confinement allows semiconductor quantum dots to exhibit size-tunable electronic and optical properties that enable a wide range of opto-electronic applications from displays, solar cells and bio-medical imaging to single-electron devices. Additional modalities such as spin and valley properties in monolayer transition metal dichalcogenides provide further degrees of freedom requisite for information processing and spintronics. In nanostructures, however, spatial confinement can cause hybridization that inhibits the robustness of these emergent properties. Here, we show that laterally-confined excitons in monolayer MoS_2_ nanodots can be created through top-down nanopatterning with controlled size tunability. Unlike chemically-exfoliated monolayer nanoparticles, the lithographically patterned monolayer semiconductor nanodots down to a radius of 15 nm exhibit the same valley polarization as in a continuous monolayer sheet. The inherited bulk spin and valley properties, the size dependence of excitonic energies, and the ability to fabricate MoS_2_ nanostructures using semiconductor-compatible processing suggest that monolayer semiconductor nanodots have potential to be multimodal building blocks of integrated optoelectronics and spintronics systems.

## Introduction

Semiconductor quantum dots (QDs) exhibit optical properties that can be tailored for diverse opto-electronic applications ranging from light emitting devices^1^, energy harvesting technologies^2^, and medical therapies^3^, to enabling rich fundamental advances in low-dimensional spintronics and quantum information processing^4–6^. In typical QDs, carrier wavefunctions are confined in all three dimensions. In the weak confinement regime, with radius *R* greater than the exciton Bohr radius *a*_B_, optical properties display size-tunable finite size effects in electronic structure, whereas the strong confinement ($R\lesssim {a}_{{\rm{B}}}$) enables discrete energy levels and fermionic exciton behavior useful for quantum devices.

**Figure 1:**
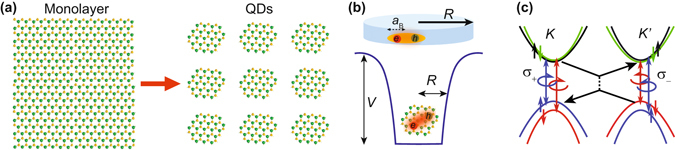
(**a**) Patterning of a 2D semiconductor into laterally-confined ML nanodots. (**b**) 2D confinement of excitons of Bohr radius *a*_B_ in a potential well of radius *R*. (**c**) Spin-orbit coupling and inversion asymmetry in ML MoS_2_ give rise to valley-specific coupling to circularly-polarized light. Confinement can potentially enhance intervalley scattering (black lines) and suppress valley polarization.

The recent interest in atomically-layered transition metal dichalcogenides (TMDs) such as MoS_2_ has revealed a class of two-dimensional (2D) semiconductors showing strong carrier confinement in one dimension while preserving a bulk-like dispersion in the 2D plane^7^. Although small TMD nanoflakes made with liquid exfoliation show blue-shifted emission for reduced lateral dimensions^8–12^, the dynamics of excitonic emission in monolayer (ML) TMD nanostructures, such as laterally-confined 2D QDs (Fig. 1a), and the emergence of defect and edge states, such as seen in graphene QDs^13^, are not systematically well-understood. Distinct from traditional semiconductors, the spatial inversion asymmetry of the hexagonal crystal structure of ML TMD semiconductors combined with large spin-orbit coupling creates degenerate valleys in the band structure with opposite spin, Berry curvature, and effective magnetic moment^14^. ML TMDs thus support several internal electronic degrees of freedom in the spin and valley pseudospin^14, 15^. The coupled spin-valley dynamics have been extensively studied both electrically and optically^16–18^, with control over the electronic spin and valley pseudospin suggested as potential for information processing^19, 20^. As observed in silicon and graphene QDs^4, 21–24^, confinement can cause intervalley coupling and valley hybridization that render the valley pseudospin to no longer be a good quantum number. The robustness of valley polarization to lateral confinement in ML TMDs remains an open fundamental question with implications for valley control schemes (Fig. 1b,c)^19, 25–27^.

Here, we create laterally-confined size-tunable atomically-thin TMD semiconductors through nanopatterning, demonstrating controlled continuous tuning of emission from bulk to a size scale down to 15 nm. Our measurements show that ML TMD nanodots in the weak confinement regime inherit the valley-selective band structure of the continuous ML while exhibiting controlled optical properties that depend on their lateral size. The semiconductor-compatible nanofabrication of ML nanostructures enables simultaneous tailoring of TMD optical properties and investigation of valley polarization in a regime of controlled lateral dimension with the possibility for integration into more complex devices for opto-electronic and valleytronics applications.

## Results

### Size-tunable optical properties

**Figure 2:**
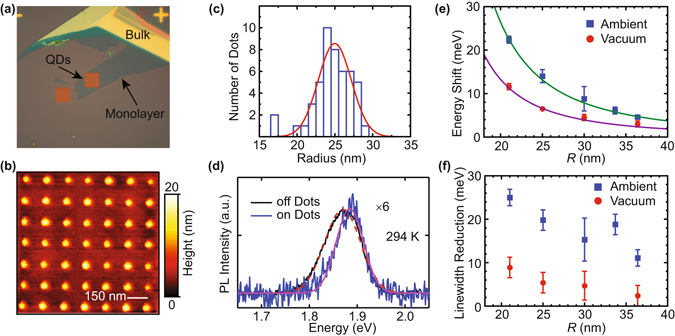
(**a**) Optical image of a nanopatterned sample. A 12 *μ*m × 12 *μ*m square of the ML MoS_2_ flake is processed into laterally confined nanodots. (**b**) AFM scan of a 1 *μ*m × 1 *μ*m region of patterned nanodots with dot spacing of 150 nm. (**c**) Nanodot size distribution from the AFM scan in (**a**). The size distribution is characterized by a normal distribution (red). For the sample shown, the average radius is *R* = 25 nm with a standard deviation of 3 nm. (**d**) PL spectra of nanodots (on dots, blue) and the continuous ML (off dots, black) from a single ML flake. (**e**) Size dependent exciton energy shift measured in ambient (blue square) and vacuum (red circle) conditions. (**f**) Linewidth reduction of nanodots measured in ambient (blue square) and in vacuum (red circle) conditions. All data in this figure are at room temperature.

The hallmark features of confinement effects are size-dependent electronic and optical properties. We develop a top-down method to fabricate size-controlled ML MoS_2_ nanodots using electron beam lithography. Semiconductor processing allows for highly accurate registration of TMD nanodot patterns with respect to other features at the 10 nm length scale, while size is controlled using etching conditions. For each sample, a partial region of a continuous monolayer flake is patterned. The un-patterned monolayer region from the same exfoliated flake is used for control measurements, as shown in Fig. 2a. Figure 2b shows an atomic force microscope (AFM) image from one sample with its size distribution shown in Fig. 2c. The patterned nanodot sizes are approximately normally distributed. Samples with an average dot radius measured as small as 20 nm were fabricated, representing the weak confinement regime in which electronic properties become size-tunable. Unlike localized defects^28–31^ or chemically prepared TMD nanoflakes^9, 10, 12^, our top-down fabrication process allows control of the position and of the lateral size of the delocalized exciton in a TMD nanodot. To differentiate from TMD QDs in a strong confinement regime^9, 10, 12^, we refer to these patterned weakly-confined monolayers as ML nanodots. Details of the fabrication, AFM size characterization, and Raman characterization of TMD nandots are given in the Methods and Supplementary Information.

The effect of lateral confinement on the MoS_2_ is measured by photoluminescence (PL) spectroscopy. A representative PL spectrum from a sample with average dot radius *R* = 25 nm (Fig. 2c) is shown in Fig. 2d. Compared to the un-patterned ML PL, the peak PL energy of the nanodots made from the same ML flake blue shifts and the linewidth (full width at half maximum) narrows. The unpatterned ML PL has comparable energy and linewidth to ML MoS_2_ exciton peaks typically identified^7^. The size-dependent energy shift of the nanodot PL relative to the continuous ML emission is evident in Fig. 2e. The energy shift is consistently smaller when measured in a vacuum chamber with pressure lower than 200 mTorr compared to in ambient conditions. The larger energy shift in air can be caused by adsorption of water molecules and oxygen^32^, which can modify monolayer electronic properties such as effective mass^33^ through doping and reduce the effective size of the confinement potential. Similar increased confinement and reduced effective size have been observed in semiconductor nanocrystal QDs while exposed to air^34–36^.

The size-dependent PL can be understood by considering excitons in the weak confinement regime. The modified eigenenergies of the exciton center-of-mass wavefunction are estimated from the envelope function approach by approximating the nearly-circular ML nanodots as a two-dimensional axisymmetric infinite potential of radius *R*. Lateral 2D confinement leads to a 1/*R* ^2^ dependence for the exciton energy levels^37, 38^. The envelope function approach is a good approximation in the weak confinement regime ($R\gg {a}_{B}$) when the exciton has large binding energy and the internal levels can be ignored^38^. This is valid for ML MoS_2_, in which *a*_*B*_ ~ 1 nm is small compared to the patterned radius in our experiment and the exciton binding energy is approximately 0.9 eV^33, 39^. In the weak confinement regime here, the center-of-mass confinement manifests as a shift in the emission rather than resolved energy levels familiar from strongly confined QDs.

Motivated by the center-of-mass energy levels of weakly confined QDs, the size-dependent PL energy shift of the patterned nanodots is characterized by a phenomenological inverse square function

1 $${E}_{ex}={E}_{0}+\frac{{\hslash }^{2}{\rho }_{0}^{2}}{2{M}_{ex}^{\ast }{(R-{\rm{\Delta }}R)}^{2}}.$$

Here, E_0_ is the PL energy without in-plane confinement (including binding energy), *ħ* is the reduced Planck’s constant, *R* − Δ*R* is the effective nanodot radius, ${\rho }_{0}\simeq 2.4048$ is the first root of the zero-order Bessel function, and ${M}_{{\rm{ex}}}^{\ast }$ is an effective mass fit parameter. Since the dot size measured by AFM is a convolution of the tip and dot geometry, and the reactive etching process or environment can alter the electronic properties of the monolayer edge, the effective nanodot radius can be several nanometers smaller than the measured size (see Supplementary Information). Measurements in both ambient and vacuum conditions show a shift of effective radius Δ*R* ~ 8 nm and are otherwise consistent with the 1/*R* ^2^ confinement behavior. The mass ${M}_{{\rm{ex}}}^{\ast }$ in vacuum (ambient) is extracted from the fit to be *M*_ex_ = 0.12 ± 0.03 *m*_0_ (0.058 ± 0.006 *m*_0_), where *m*_0_ is the free electron mass. The difference between the ambient and vacuum parameters suggests that the nanodots are influenced by adsorption from the environment^32^, which would be expected to be increasingly significant in nanostructures with increased surface-to-volume ratio. Photocarrier relaxation processes from the non-equilibrium optical excitation impact the observed emission energy shift. The energy scaling is also influenced by electron-electron interactions, doping, strain, and dielectric environment, all of which are known to influence monolayer electronic properties^33, 39–43^. Additional discussion of the energy shift is provided in the Supplementary Information.

The PL linewidth Γ decreases with confinement compared to the linewidth in un-patterned MoS_2_ ML as shown in Fig. 2f. The linewidth reduction is larger for smaller nanodots, with the narrowest linewidth measured to be about 25 meV smaller compared to a typical MoS_2_ ML PL linewidth. The reduction measured in vacuum is smaller compared to that in ambient conditions. Although the difference between vacuum and ambient conditions warrants further investigation, the 1/*R* ^2^ dependence of the exciton energy shift and narrower PL linewidth demonstrate controlled lateral size-dependent effects in a TMD monolayer.

### Temperature dynamics of laterally confined nanodots

**Figure 3:**
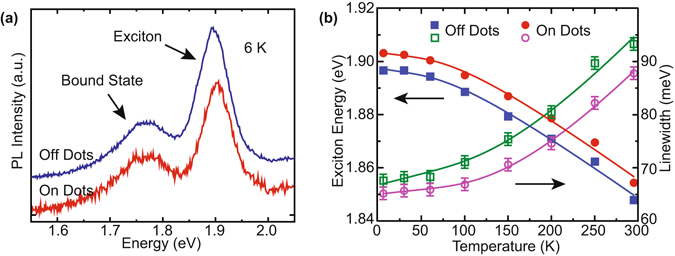
PL spectra characterization. (**a**) The PL of the nanodots (red) and continuous ML (blue) at 6 K both show features of excitonic and low-energy bound state emission. (**b**) Temperature dependence of the exciton energy *E*_ex_ (solid) and linewidth Γ (empty) for nanodots (circles) and ML (squares). The lines are fits to the Manoogian-Wooley equation^62^ and Eq. (2), respectively.

Figure 3a shows low-temperature PL spectra collected when the excitation is either on the dots or the continuous ML control region. A peak at about 1.9 eV and a low-energy state at 1.77 eV are evident. The peak energy and lineshape are both consistent with ML exciton emission reported in other studies^44–46^. ML MoS_2_ is well-known to exhibit lower-energy bound state luminescence at low temperatures^47, 48^. The lateral confinement in our nanodots does not modify the energy of this bound state PL at 1.77 eV, indicating that the bound state is formed by localized defect states^49^ rather than free band excitons. Temperature dependence of the emission energy and linewidth are shown in Fig. 3b. The energy difference between ML and nanodots is independent of temperature, consistent with the shift arising from a size-dependent confinement energy rather than thermally-activated defect doping or redistribution of trion and exciton populations. Additional discussion of the PL behavior appears in the Supplementary Information.

The linewidth Γ provides information on homogeneous broadening and inhomogeneity of the material^43, 50^. At low temperatures, the homogeneous linewidth is dominated by the acoustic (AC) phonon dephasing that scatters excitons to intraband states. At higher temperatures, the longitudinal optical (LO) phonon interaction scatters excitons to both bound and continuum states, with contribution to the linewidth proportional to LO phonon number^50, 51^. Neglecting any screening and doping effects specific to ML materials, the temperature dependence of Γ for excitons in a continuous dispersion band can be expressed as

2 $${\rm{\Gamma }}(T)={{\rm{\Gamma }}}_{0}+{\gamma }_{AC}T+\frac{{\gamma }_{LO}}{{e}^{{E}_{LO}/{k}_{B}T}-1}$$

where Γ_0_ includes the inhomogeneous and radiative linewidth, *γ*_*AC*_ and *γ*_*LO*_ are the exciton-AC and exciton-LO phonon coupling strengths, and *E*_*LO*_ is the LO phonon energy.

Figure 3b shows fits of the linewidth of continuous ML and nanodots to Eq. (2). As with emission energy, the temperature dynamics of Γ are similar for both ML and nanodot regions, but Γ for the nanodots is somewhat reduced (details of the fits are in the Supplementary Information). Inhomogeneous broadening in Γ_0_ is expected to be much larger than the small intrinsic linewidth^43^ and will dominate the overall linewidth Γ. Γ_0_ is similar in ML and nanofalkes (Γ_0_ is 67 ± 1 meV and 65 ± 1 meV, respectively), suggesting that unintentional doping from fabrication is not influencing emission. Rather, the inhomogeneities are caused by local disorder potentials from lattice, surface, and substrate defects that remain significant across the wavefunction scale. Broad PL linewidths have also been reported in single chemically-exfoliated nanoflakes in a stronger confinement regime, so confinement with controlled nanopatterning in the regime here is not expected to narrow the low temperature exciton linewidth^12^.

The change of the PL linewidth of the nanodot ensemble is determined by two competing mechanisms. Confinement narrows the homogeneous linewidth by reducing the available acoustic phonon states, whereas the inhomogeneous size distribution broadens the linewidth. We indeed find that the *γ*_*AC*_ in the nanodots (12 *μ*eV/K) is smaller than that in the monolayer (42 *μ*eV/K). The 10% width of the nanodot size distributions (Fig. 2b) introduces a 20% variation in confinement energy due to the 1/*R* ^2^ dependence. Because of the small energy shifts in this regime, the size distribution only causes less than 2 meV of broadening. This interpretation is supported by measurement of PL from a few WSe_2_ nanodots, which also shows a similarly broad linewidth (see Supplementary Information). The reduction of Γ at high temperatures implies that the confinement effects dominate the inhomogeneous broadening introduced by fabrication, consistent with observations in other confined systems such as GaAs^51–53^.

Similar lithographically-defined laterally confined structures have been etched from semiconductor quantum wells. In contrast to the monolayer TMD nandots, the etched quantum wells typically exhibit non-radiative PL broadening^54, 55^. This non-radiative broadening originates from defects at the etched sidewall edges. The weakly confined excitons of the TMD, with Bohr radius *a*_B_ ~ 1 nm, are less sensitive to edge defects and surface chemistry than in conventional quantum wells with larger Bohr radius typically on the order of 10 nm; phonon processes dominate over edge effects in the TMD nanodots. Smaller chemically exfoliated TMD nanoflakes in the strong confinement regime do exhibit non-radiative broadening likely from exciton coupling to surface defects similar to etched quantum wells^12^.

### Valley polarization in weakly confined monolayer MoS_2_ nanodots

The spin-orbit coupling and inversion asymmetry in the TMD monolayer allow selective optical excitation of different valleys using circularly polarized light, which can be detected as circularly-polarized PL emission^14, 16, 17^. Valley hybridization from intervalley scattering can reduce emission polarization, as would be expected at elevated temperatures or near monolayer edges^16, 44^. To investigate the impact of quantum confinement on valley hybridization in MoS_2_ nanodots, we measure polarized PL.

**Figure 4:**
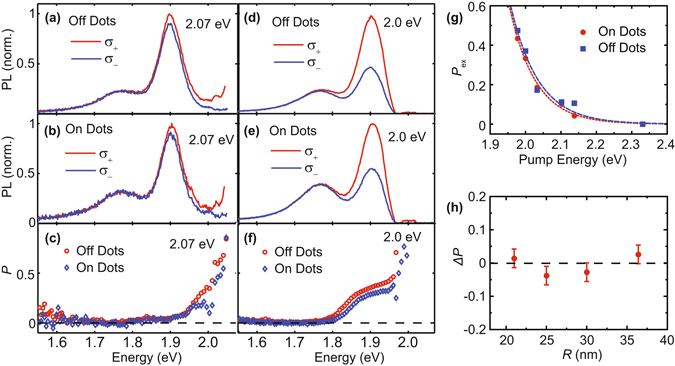
Valley polarization characterization from a continuous ML and nanodots. (**a**,**b**) Circularly-polarized emission with pump energy of 2.07 eV. (**c**) Polarization *P* of PL with pump energy of 2.07 eV. (**d**,**e**) Circularly polarized PL with pump energy of 2.00 eV. (**f**) Polarization *P* with pump energy 2.00 eV. (**g**) Pump energy dependence of the continuous ML (off dots) and nanodots (on dots). The characteristic energy exponential decay scale is *ξ* = 74 meV for nanodots (red) and *ξ* = 79 meV for a continuous ML (blue). (**h**) The negligible change in PL circular polarization with confinement is independent of nanodot size. Polarization is measured with pump energy at 2.00 eV for (**g**,**h**). PL in this figure is collected at 6 K.

Figure 4 shows polarized PL measured on and off nanodots with *R* = 25 nm using two different pump energies, 2.07 eV and 2.00 eV. The spectrally-resolved PL polarization is defined by

3 $$P(\lambda )=\frac{{I}_{+}-{I}_{-}}{{I}_{+}+{I}_{-}}$$

where *I*_+_ and *I*_−_ are the right (*σ*_+_) and left (*σ*_−_) circular polarization-resolved PL spectral intensities. The nanodot emission exhibits similar polarization to that of the ML for each pump energy as shown in Fig. 4c,f. The slightly smaller nominal nanodot polarization is due to the higher relative intensity of the tail from the unpolarized low-energy bound state at 1.77 eV and the background. After subtracting a fit to the bound state PL and background, we observe almost identical (within our measurement accuracy) pump-dependent polarization of nanodot and ML emission as shown in Fig. 4g, where the polarization is calculated similarly to Eq. (3) but using the integrated intensities of the exciton PL peak. For both nanodots and ML, the polarization increases for pump energy closer to the exciton resonance. The pump energy dependence (Fig. 4g) can be characterized by a phenomenological exponential curve $P\sim \exp \,[-({E}_{{\rm{pump}}}-{E}_{{\rm{ex}}})/\xi ]$, where *E*_pump_ is the pump energy, *E*_ex_ is the exciton energy, and *ξ* describes the energy decay scale. The fit to nanodots and ML show similar decay scale *ξ* = 0.074 ± 0.006 (0.079 ± 0.013) eV. The pump energy dependence of *P* is similar to that previously reported for MoS_2_ and associated with acoustic phonon-driven intervalley scattering^45^. Figure 4h shows the difference of emission polarization between nanodots and continuous ML sheet for varying nanodot size. Within the measurement precision, nearly unchanged polarization is observed across all the nanodot sizes.

The persistence of valley polarization in weakly confined ML TMD quantum dots is expected from theoretical predictions^25^. Despite the strong dependence of intervalley scattering on confinement potential, the upper bound of the intervalley coupling in ML MoS_2_ dots is 0.1 meV for small sizes (20 nm in diameter)^25^ which is negligible compared to the energy difference between the same spin states in different valleys, for both electrons (several meV)^56^ and holes (~150 meV)^14, 56^. Therefore, valley polarization is expected to be only weakly perturbed in this size nanodot. Although in graphene nanoribbons a width of 30 nm is enough to create tens of meV valley splitting^4^, excitons in TMD nanodots exhibit weaker wavefunctions near the boundaries where intervalley coupling is significant, leading to correspondingly weaker valley hybridization^25^. Our observation of valley-polarized emission in quantum confined ML nanodots confirms the theoretical prediction of Liu^25^ that intervalley scattering is negligibly increased in ML MoS_2_ dots with radii as low as 15 nm.

## Discussion

The evolution of valley polarization in TMD nanostructures with lateral confinement has not been explored in previous experiments. Polarized PL is not reported from highly confined monolayer nanoflakes^8–12^. Our work demonstrates that lateral confinement does not measurably increase intervalley scattering in ML MoS_2_ dots down to the size of 15 nm, illuminating optical behavior in this intermediate size-dependent regime. If the valley polarization is unperturbed by even stronger lateral confinement, ML TMD nanostructures might be a viable platform for exploring the valley degrees of freedom of a single electron^20^. The techniques required to achieve strong lateral confinement in smaller patterned monolayer nanostructures and the new quantum phenomena and edge effects that would arise in this regime^19, 26, 27^ are beyond the scope of this work.

The controlled size-dependence of low-dimensional excitons is an important consequence of lateral confinement useful for many applications of semiconductor nanomaterials and heterostructures^57–60^. Our nanofabrication approach enables size-tunable lateral confinement in ML MoS_2_ nanodots using a controllable top-down semiconductor-compatible fabrication process. The size-dependent, but temperature independent, energy shift indicates that its origin is not unintentional edge or fabrication doping. Recently, quantum emitters in 2D TMDs have been demonstrated from defect states in monolayer WSe_2_^28–31^. Although interesting for quantum information applications, these randomly distributed localized states do not exhibit size-dependent excitonic properties indicative of lateral confinement or the valley polarization of excitons in the monolayer TMD band structure. Distinct from these defect emitters, our results demonstrate controlled size-dependent confinement of delocalized band carriers. Since excitons in monolayer TMDs have very large binding energies and small Bohr radii (*a*_*B*_ ~ 1 nm), it is challenging to reach a strong confinement regime with an anharmonic spectrum and antibunched emission using top-down processing.

Systematic control of size in TMD nanodots allows exploration of the optical properties of isolated ML nanostructures. Our work demonstrating size-tunable lateral confinement is a first step toward control of three-dimensional confinement in monolayer semiconductors. The inheritance of ML valley properties in ML semiconductor nanodots with size-controlled exciton energies suggests that TMD ML nanostructures are a potential platform for harnessing the valley degree of freedom of a single electron^19, 20, 25^. Extending semiconductor-compatible nanofabrication of laterally-confined ML nanodots with controlled size-dependent features can help to realize low-dimensional building blocks for integrated opto-electronic systems^11^ based on spin and valley.

## Methods

### Sample preparation

ML MoS_2_ flakes are obtained from a bulk crystal (SPI Supplies) using mechanical exfoliation. The flakes are dry transferred onto a SiO_2_/Si substrate with pre-written alignment marks^61^. Identification of ML MoS_2_ is done using atomic force microscopy (AFM), optical contrast, and PL spectroscopy following documented procedures^61^. Samples are annealed in an Ar/H_2_ environment at 400 ºC before patterning with electron beam lithography and reactive ion etching using CHF_3_/O_2_ gas. The resist is not removed at the end of processing to prevent destruction of the ML nanodots. Additional sample fabrication and material characterization details can be found in the Supplementary Information.

### Optical measurement

PL characterization was performed with a 532 nm semiconductor laser, and near-resonant circularly-polarized PL is measured with a tunable pulsed femotosecond optical parametric oscillator whose linewidth was narrowed to approximately 1 nm using a double-grating system. The pump laser is focused through a 100× long-working distance objective with numerical aperture of 0.65 and a spot size near 1 *μ*m. The pump power is kept less than 40 *μ*W to suppress heating effects. The sample is mounted in a closed-cycle cryostat capable of reaching temperatures between 4 K and 350 K. The objective can be scanned across the sample with sub-micron resolution. The PL is collected through the same objective and analyzed by a spectrometer with a CCD. Energy and linewidth shifts for nanodots are reported relative to the continuous ML control region from the same exfoliated flake. The low areal density of the sparse array pattern precludes absorption measurements of the processed monolayer nanodots. For circularly-polarized PL, the samples are stabilized at 6 K. The pump polarization is controlled using a linear polarizer and a liquid crystal retarder. The emitted PL is analyzed using a quarter waveplate and a linear polarizer before being coupled into the spectrometer. The polarization of the pump laser is measured to be greater than 99.5% with this apparatus. Reported best-fit parameters are obtained from weighted least-squares fitting with parameter uncertainties estimated using resampling.

### Data availability

The data that support the plots within this paper and other findings of this study are available from the corresponding author upon reasonable request.

## Electronic supplementary material

Supplementary Information
