# Supplementary material for: Size-tunable Lateral Confinement in Monolayer Semiconductors
Source: Sci Rep. 2017 Jun 12;7:3324. doi: 10.1038/s41598-017-03594-z (PMC5468254; doi:10.1038/s41598-017-03594-z)
Supplement: Supplementary file 1 — Supplementary Information [file 41598_2017_3594_MOESM1_ESM.pdf]

Supplementary Material for:
Size-tunable Lateral Confinement in Monolayer Semiconductors

Guohua Wei[1], David A. Czaplewski[2], Erik J. Lenferink[3], Teodor K. Stanev[3], Il Woong Jung[2], and Nathaniel P. Stern[1,3]

[1]*Applied Physics Program, Northwestern University, Evanston, IL 60208, USA*

[2]*Center for Nanoscale Materials, Argonne National Laboratory, Argonne, IL 60439, USA*

[3]*Department of Physics and Astronomy, Northwestern University, Evanston, IL 60208, USA*

Fabrication

Monolayer MoS_2 flakes are obtained using mechanical exfoliation with the Scotch-tape technique from a bulk crystal (SPI Supplies). The flakes are dry transferred onto a SiO_2/Si substrate with pre-written alignment marks [1, 2]. Identification of monolayer MoS_2 is routinely done using atomic force microscopy (AFM), optical contrast, and photoluminescence spectroscopy as described previously [2]. Devices are annealed in an Ar/H_2 environment before patterning with electron beam (e-beam) lithography.

Nanodot patterning is performed using a JEOL 9300 electron beam lithography system operating at 100 kV. Pattern writing is only performed on part of each flake to enable control measurements on the unprocessed continuous monolayer from the same MoS_2 flake (Fig. S1). Typical optical images of a device are shown in Fig. S1. To reduce exposure damage to the monolayer by the electron beam, larger size rectangular regions with side length $\sim$ 80 nm are first patterned by e-beam lithography using positive resist (GL2000) with cold development, illustrated in Fig. S2. A reactive ion etch (RIE) is performed following the e-beam patterning process. The RIE conditions are 20/10 sccm of CHF_3/O_2 gas flow with 30 W RF power at a pressure of 50 mTorr. The etching time is 10 − 40 seconds depending on the desired size. Fig. S1c,d shows the AFM images of one sample. After RIE etching for 20 s, the nanodot size is clearly smaller than the initial pattern, enabling control of the final effective size. Etching for 10 s is sufficient to remove the monolayer in the region that has been exposed; longer etching times reduce the nanodot size from the original pattern while rounding the corners. The resist is not removed at the end of processing to prevent destruction of the monolayer nanodots. The patterning process is optimized for arrays. The RIE chemical process becomes less reliable when the dots are more sparse, making isolation of a single nanodot challenging with the current fabrication process.

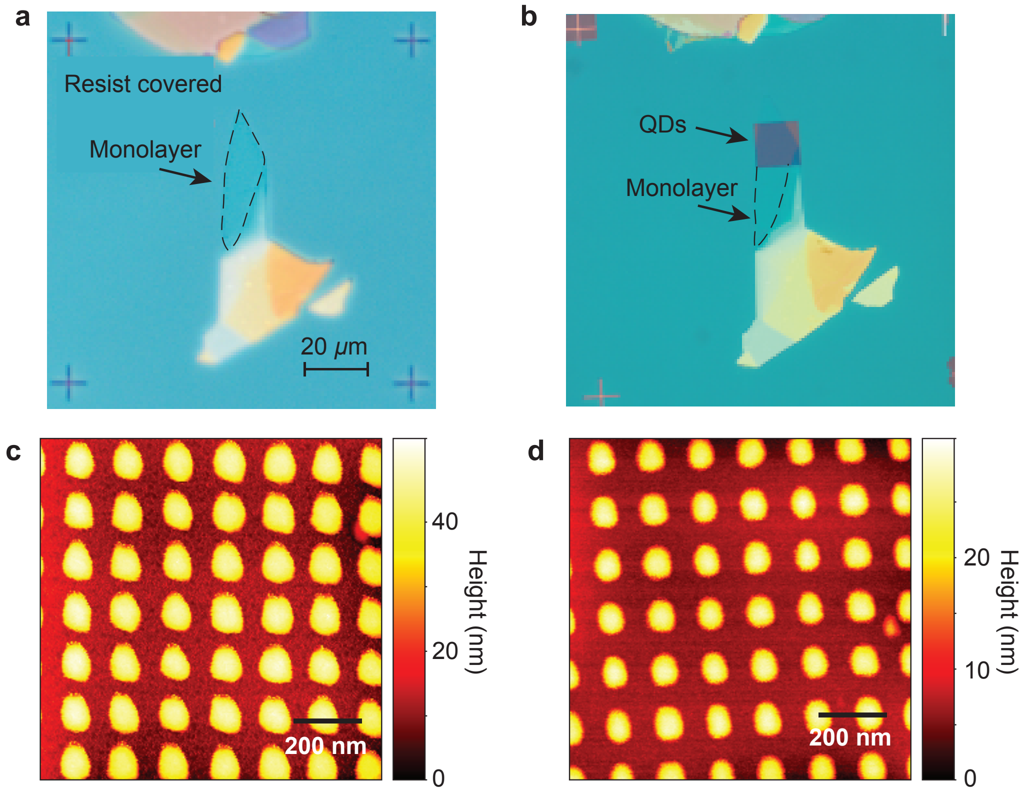

Figure S1: Optical and AFM images illustrating the fabrication process. **a**, **b** Optical images of the monolayer flake before (**a**) and after (**b**) fabrication. The blue color is the resist layer that covers the top of the sample except in the e-beam patterning region (square). The outline of the monolayer shows that only a part of the monolayer flake has been patterned into nanodots. **c**,**d** AFM images of nanodots before (**c**) and after (**d**) RIE etching. The 40 nm height reading is because the resist is not removed from the top of the nanodots.

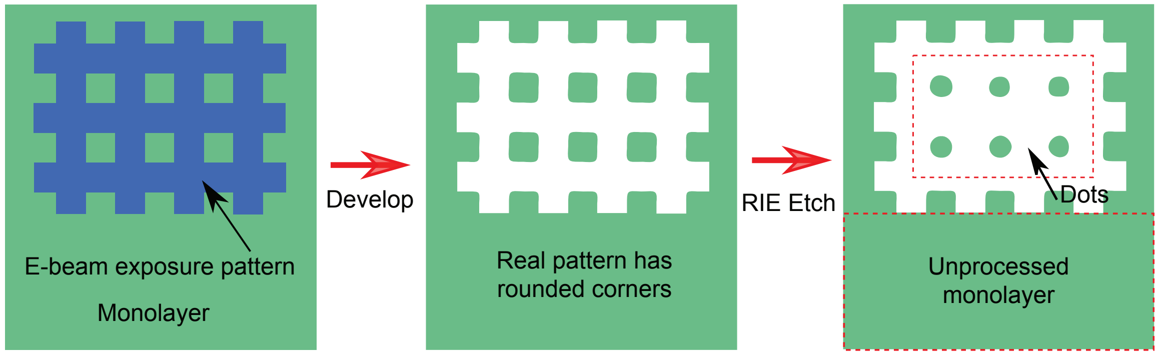

Figure S2: Illustration of the e-beam lithography process. The arrows show the process steps. A monolayer flake is patterned with lines that criss-cross over a region of the flake. Because of the small size, the square pattern exhibits rounded corners after developing. These large regions with radius of about 40 nm are etched by RIE to make even smaller monolayer dots. The exposed monolayer is removed during this process.

Size characterization

Nanodot size is characterized by atomic force microscopy (AFM) in noncontact mode. The size distribution used in the main text is obtained from image processing. The nearly-circular shapes are modelled as circular dots with diameter D, so that $\pi D^2/4$ equals to the area of each dot from the AFM scan. The actual size of a monolayer nanodot differs from that measured directly from image processing of the AFM data. Since the AFM tip has a finite size, the lateral resolution of an AFM scan depends on the tip geometry. The real AFM profile of the nanodots is a convolution of the AFM tip profile and the sample geometry that depends on the side wall angle β shown in Fig. S3 [3]. Directional asymmetry in the side wall angle is observed due to the scan direction asymmetry, but this does not impact the conclusions of the typical geometric size correction described here.

In our nanodots topped by a photoresist layer, β is always smaller than 60°. Since the AFM tip angle is normally smaller than 30°, the tip-sample convolution is determined by the rounding of the AFM tip illustrated in Fig. S3b, in which the measured topography follows the blue curve while the real surface is the green curve. The measured full width at half height radius $W_{\mathrm{FWHM}}/2$ differs from the actual radius $W/2$ by a geometric correction $\Delta = W_{\mathrm{FWHM}}/2 - W/2$. Assuming the AFM tip is rounded with radius of curvature R_{tip}, the measured tip path results in a geometric correction to the true dot radius of $\Delta = R_{\mathrm{tip}} \tan(\beta/2)$. For a typical fresh AFM tip with $R_{\mathrm{tip}} \sim 10$ nm, the correction Δ is around $3 - 5$ nm. Since the AFM tip broadens with usage, this correction should be a lower limit on the expected geometric size correction.

As described in the text and references, chemical adsorption at the edge can result in reduction of the effective nanodot size. $\Delta R \sim 8$ nm is extracted from the energy dependence, which is reasonable due to the combined geometric and chemical mechanisms.

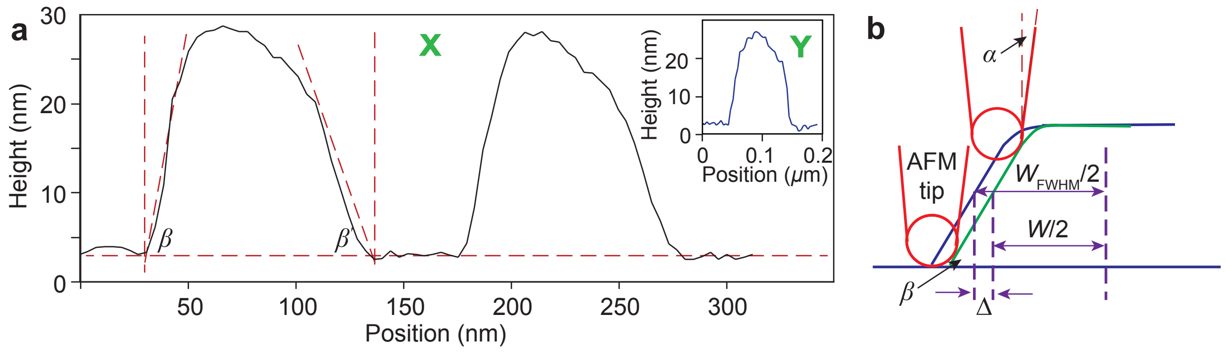

Figure S3: AFM tip-sample convolution. **a**, The AFM height profile of nanodots scanned along direction X. The inset shows the perpendicular direction Y. β and β' are direction-dependent side wall angles. Slightly asymmetric angles are measured in some AFM profiles due to the directional scanning, but this disappears under symmetric conditions (as in the Y data). **b**, The geometric convolution (blue) of the AFM tip radius R_{tip} with the actual side profile (green).

Raman spectra of monolayer nanodots

Raman spectroscopy is a versatile method for 2D material characterization because it provides a convenient and nondestructive optical probe of crystal structure and response. The Raman spectrum sensitivity to structural changes has been applied to study layer and strain-dependent properties of 2D materials [4, 5, 6, 7]. Raman spectroscopy has also been widely used for studying nanostructured materials from nanocrystals [8, 9, 10] to graphene nanoribbons [11, 12] and even the edge chirality of graphene [13]. Shown in Fig. S4, when applied to lithographically patterned MoS_2 nanodots, Raman spectroscopy shows two modes similar to those in an unprocessed monolayer. The intensity of the nanodots is much weaker expected for the low areal density. The Raman frequency is blue shifted slightly, which could be a result of the phonon confinement effect [6] that allows phonons with larger wavevector to be involved in the Raman scattering process. The confinement effect typically broadens the linewidth [10], but we observe the opposite effect. We also did not see a clear trend comparing nanodots with different sizes. Another possible explanation of these features is the different strain between the monolayer and processed nanodots [7]. Although the origin of the Raman shift and linewidth change requires further investigation, our measurements clearly do not reveal edge modes in the Raman spectra as one may see when defects and edge effects dominate [6].

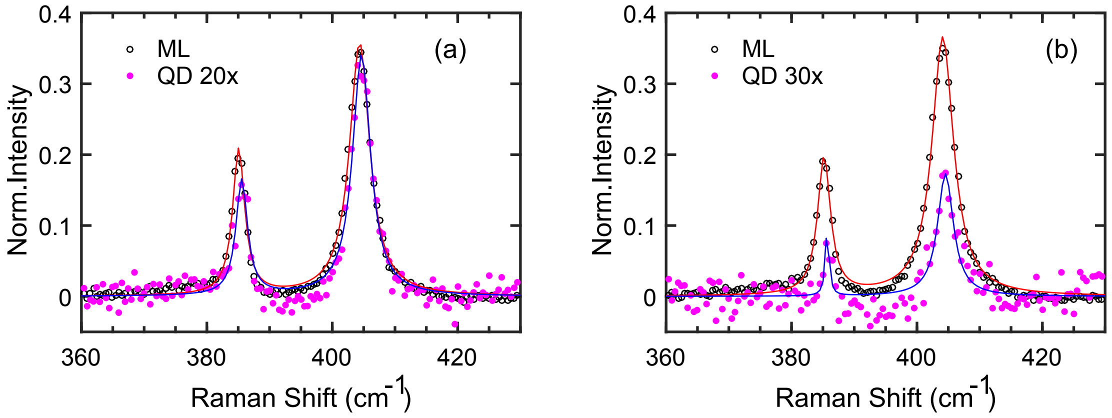

Figure S4: Raman spectra of monolayer MoS_2 nanodots with radius of (a) 25 nm and (b) 20 nm compared to the unprocessed monolayer. The nanodot spectra are magnified by 20 and 30 times respectively.

Fitting of temperature dependent data

The temperature dependence of the PL peak energy for the nanodots and the continuous ML is typical for semiconductors and can both be fit by the Manoogian-Woolley equation [14].

The main text outlines the phonon-scattering model for the temperature dependent linewidth. Here, we give the numerical fit results of this various parameters of this model for both continuous monolayers and patterned nanodots. To perform the fit, the LO phonon energy E_{LO} is fixed to be 48 meV [15]. The best fit parameters for the inhomogeneous linewidth are $\Gamma_{\mathrm{inh}} = 65.0 \pm 0.9$ meV for nanodots and $\Gamma_{\mathrm{inh}} = 66.8 \pm 0.9$ meV for the continuous monolayer. The slightly smaller Γ_{inh} for nanodots indicates that in the absence of phonon interactions, nanodots exhibit smaller inhomogeneous broadening despite their size distribution. Linewidth reduction has also been observed in other weakly confined systems [16]. We find the γ_{LO} to be 86 ± 18 meV and 101 ± 18 meV in ML and nanodots respectively. First principle calculation predicts γ_{LO} to be less than 100 meV [15], consistent with our measurement. The fitting gives exciton-acoustic phonon interaction γ_{AC} in ML MoS_2 as 42 μeV/K, which is comparable to the value (60 μeV/K) in ML WSe_2 measured by two-dimensional Fourier transform spectroscopy [17].

Origin of the PL and possibility of trions

As-exfoliated monolayer MoS_2 typically exhibits n-type doping properties, and its PL emission can include both neutral exciton and negatively charged exciton (trion) character [18, 19, 20]. Trion emission is often dominant in MoS_2 at low temperature.

The monolayers in the present experiment do not show evidence of resolved multiple emission peaks. Although a contribution of trions to the nanodot PL is possible, we expect it to be small for ungated MoS_2 on an SiO_2 substrate [21] and the energy shift measured in the experiment is likely dominated by excitonic emission.

Electrical gating and charges can change the relative importance of neutral excitons and trions. Unintentional doping near the terminated edges of the patterned monolayers could lead to enhanced trion emission. However, trions have lower emission energy than the exciton. In the experiment, lateral confinement leads to an observed increase in emission energy, so fabrication is not likely enhancing trion emission. Rather, a more likely explanation of the emission in our experiment is related to the protective resist layer. Resist residue is known to p-dope graphene [22], which we have similarly observed with MoS_2. The resist on our samples is likely p-doping the monolayer, thereby suppressing trion generation in all samples. Therefore, the size-dependent effects here

likely are not dominated by trion emission.

The size-dependent, but temperature independent, energy shift suggests that its origin is a single state and that the energy shift does not arise from redistribution of trion and exciton populations caused by unintentional edge or fabrication doping. The temperature insensitivity is highly suggestive of a purely size-dependent effect. As shown in this Supplementary Information, we have also observed similar blue shifting and narrowing linewidth of the patterned nanodot PL from monolayer WSe_2, which has no trion signature at room temperature due to p-type doping [23, 24, 25].

Size-dependent energy shifts

Rough understanding of the vacuum effective mass fit parameter is obtained by considering the energy spectrum of a weakly confined quantum dot (QD) or nanodot. Because the center-of-mass energy levels $E_{\mathrm{ex,n}}$ are more closely spaced than the emission linewidth, a simple ground state model (Eq. (1) from main text with $M^*_{\mathrm{ex}} = M_{\mathrm{ex}} = m_e + m_h$) would not be expected to describe the size dependence. Rather, the emitting energy level distribution of weakly-confined systems is non-thermal [26]. Excited energy levels, with their significantly larger energy shifts, will contribute and the overall energy shift can be larger, manifesting as a reduced M^*_{ex} in a fit to Eq. (1). As expected, the fit parameter M^*_{ex} is smaller than that implied by a single ground state level with exciton total mass $M_{\mathrm{ex}} \simeq 0.8\, m_0$. The role of the non-equilibrium emission from weakly confined center-of-mass energy levels is discussed in the next section. The energy scaling is also influenced by electron-electron interactions, electron-phonon interactions, doping, strain, and dielectric environment, all of which are known to influence monolayer electronic properties [27, 28, 29, 30, 31, 17]. Our ML samples, sandwiched between the SiO_2 substrate and the e-beam resist, would be sensitive to these factors. The increased Coulomb interaction, edge effects, and enhanced screening in layered materials present opportunities for further theoretical work to better understand emission in monolayer nanostructures in the weak confinement regime.

Toy model for size-dependent energy shifts

In the main text, it is shown experimentally that the emission energy of ML TMDs depends on the lateral size with a shift scale of a few meV. The shift is characterized by an effective mass $M^*_{\rm ex}$ which is defined in such a way to compare to the predicted shift from a simple ground state exciton confinement model, which would be parameterized by the exciton total mass $M_{\rm ex} = m_e + m_h \approx 0.8 m_0$ in MoS_2. The excitonic properties of the patterned ML nanodots are expected to be significantly influenced by a variety of environmental properties not measurable in this experiment, but a simple non-equilibrium relaxation model with weak confinement can reproduce the order of magnitude of the observed energy shift despite the ML nanodots being much larger than the Bohr radius of the MoS_2 excitons.

In the exciton weak confinement regime, the spatial wavefunction of tightly-bound exciton states is given by the center-of-mass (CM) wavefunction. A simple 2D axisymmetric potential leads to a spectrum of energy levels for center-of-mass (CM) states $|n\rangle$ labeled by integer n:

$$E_{\rm ex,n} = \frac{\hbar^2 \rho_{0,n}^2}{2 M_{\rm ex} R^2} \tag{S1}$$

where $\rho_{0,n}$ is the $n^{\rm th}$ root of the zero-order Bessel function, $M_{\rm ex} = m_e + m_h$ is the exciton total mass, and R is the radius. These levels have a spatial distribution given by the zero-order Bessel functions of the first kind, and are composed of the CM quasimomentum states labeled by $\vec{K}$. Internal levels of the tightly-bound exciton are ignored by this treatment. The phenomenological Eq. 1 of the main text matches the $n = 1$ level energy when $M^*_{\rm ex} = M_{\rm ex}$ (and the effective radius is used).

Since these levels are generally closely spaced relative to the linewidths, the multi-state emission must be considered. Here we present a simple toy model that demonstrates how optical pumping of the ensemble of center-of-mass energy levels can lead to an energy shift of the broadened PL spectrum larger than that of a single energy level.

Optical excitation of the sub-wavelength nanodots is uniform over their surface, and the local hot exciton population density generated by this excitation is also uniform. Fast relaxation of excitons by LO phonons will populate the closely-space levels before exciton recombination. The emission dynamics will be determined by evolution of this non-equilibrium exciton distribution. This spatially uniform exciton density can be expanded in the spatial CM wavefunction basis $|n\rangle$. The overlap of the $|\vec{K} = 0\rangle$ state of uniform exciton density with the CM wavefunctions is

$$\langle k = 0 | n \rangle = \frac{2}{\rho_{0,n}} \tag{S2}$$

For a continuous ML where all levels are degenerate, each of these states would be populated

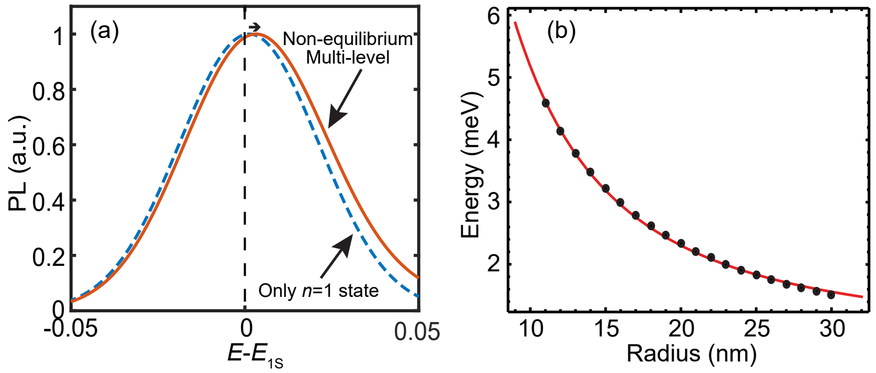

Figure S5: Multi-level PL calculation. **a**, Assuming a Gaussian inhomogeneously broadened linewidth and non-equilibrium emission weights determined by the spatial overlap of CM wavefunctions with the uniform excitation, the PL line will shift to higher energy from that of only the $n = 1$ CM state. **b**, The size-dependent shift from such a model can be fit with a R^{-2} function with effective mass M^*_{ex} larger than the exciton total mass.

across the flake to give a spatially uniform emission. In a nanodot, however, the CM states are not degenerate, and each $|n\rangle$ state pumped by the uniform excitation will emit PL with a slightly different energy. Since the higher n states have a more pronounced energy shift, the shift of the full PL line will be determined by the relative populations of these spatial CM wavefunctions.

To demonstrate this multi-level effect in a nanodot, we assume each state emits a broadened PL line with a weight given by its spectral weight from the excitation (the square of its overlap). Fig. S5a,b shows a sample calculated emission and size-dependent energy shift assuming a Guassian lineshape for each emitting level. The overall shift of a Gaussian fit function is characterized by $M^*_{\text{ex}} = 0.26m_0$. This is only a factor of 2 from the experimental fit, with no environmental effects, screening, or dynamics taken into account.

Upon optical excitation, the excitons are in a highly non-equilibrium configuration. The above toy model assumes that the excited CM states do not relax before emission to preserve the non-equilibrium energy distribution. The ability for the exciton population to thermalize depends on the redistribution of the uniform spatial exciton density into the lower energy wavefunctions. Although the wavefunctions are orthogonal ($\langle n|m\rangle = 0$), CM states are coupled by the phonon scattering potential V_{ph} ($\langle n|V_{\text{ph}}|m\rangle \neq 0$), which is dominated by acoustic phonons for small energy separations.

At low temperature, homogeneous TMD emission is dominated by radiative broadening [17], and non-thermal exciton emission is expected even from an unconfined ML [32]. At elevated temperatures, phonon scattering becomes much more significant (evidenced here by the dephasing in Fig. 3b of the main text) and ML emission can be thermal [32]. In a dot, however, confinement of CM wavefunctions reduces the phonon-induced energy relaxation due to the narrow density of states. A calculation of acoustic phonon relaxation between the ground and first excited CM energy

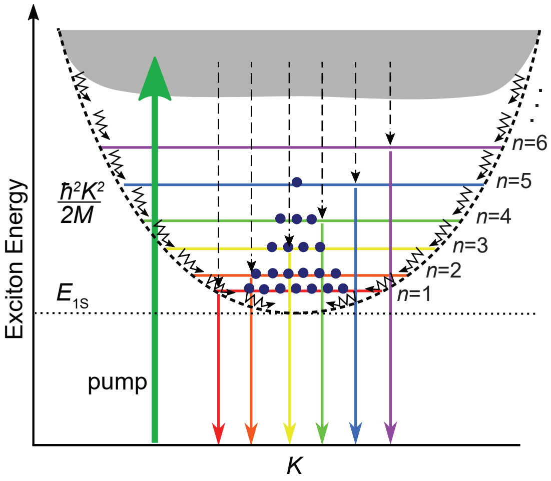

Figure S6: Schematic of the toy emission model. Optical pumping results in fast occupation of the CM levels by optical phonon scattering with distribution given by their spatial overlap. At low temperature, this non-thermal exciton population will emit quickly before relaxation [32]. For a free 1S exciton, the CM dispersion is continuous (black dotted line) and acoustic phonon scattering leads to fast relaxation and thermalization of the CM states. In nanodots with discrete CM energy separations, acoustic phonon scattering with the quantized levels is suppressed, and the emission can remain non-thermal even at high temperatures [26].

level in a 20 nm nanodot using phonon parameters for MoS_2 [15] shows that the relaxation rate is reduced by several orders of magnitude to the nanosecond scale at room temperature. The reduction of acoustic phonon scattering and the corresponding non-thermal CM energy level emission is well-known in weakly-confined semiconductor heterostructures [26, 33, 34]. Under these conditions, which are distinct from a continuous ML, the weakly-confined exciton population must be treated as a non-equilibrium system, and the closely-spaced energy levels will not have the time to thermalize before exciton emission even at room temperature.

Although not intended to be precise, the non-equilibrium multi-level energy shift toy model presented here and summarized in Fig. S6 qualitatively justifies the temperature independent features of the confinement shift and the reduced M^*_{ex} fit parameter. For more realistic quantitative results, the dielectric environment of substrate and capping layer, charge doping, trion effects, hot excitons, and phonon-coupled exciton relaxation rate dynamics would need to be considered, which are beyond the scope of the present measurements.

Device Stability

Unlike nanocrystal QDs and colloidal QD solids, which generally have very short lifetime even in inert environments [35, 36, 37, 38, 39], monolayer MoS_2 nanodots are found to perform consistently over the period of time that we have measured them. Fig. S7 shows PL measurements from the R = 20 nm nanodot sample separated by over two months. The red PL spectra are measured two months after the blue spectra. The PL peak energy and linewidth agree well in both measurements. Similar preservation of performance over many months was also observed from other samples. After up to four months of storing a nanodot device in a desiccator, PL quenching is not observed and the exciton energy shift is reproducible. The relatively long lifetime can be a result of the resist on top of the material's surface. Removal of the protective resist has not yet been explored.

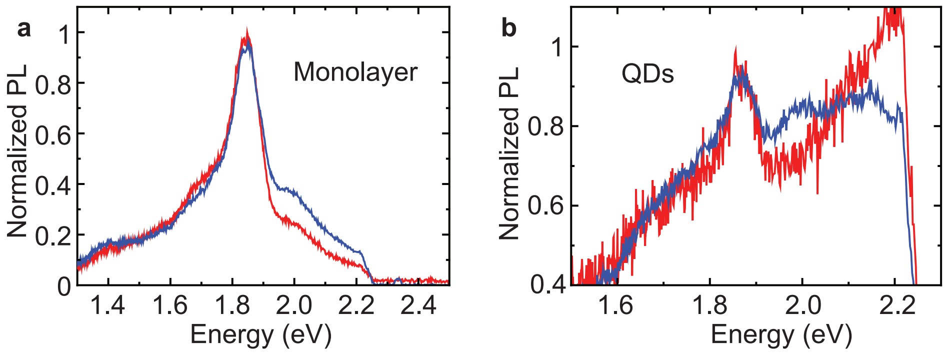

Figure S7: Device stability over two months. **a**, The red PL spectrum is measured two months after the blue PL spectrum from the same monolayer. **b**, The same measurements for patterned nanodots.

WSe_2 monolayer nanodots

To demonstrate the applicability of our processing methods to a broader range of TMD monolayers, we applied the same procedures to exfoliated monolayer WSe_2. Fig. S8 shows the PL spectra of a monolayer WSe_2 device measured from an unpatterned region (off dots) and from a patterned nanodot region (on dots). The nanodots have radius $R \sim 30$ nm. A blue shift (~8 meV) of the PL peak energy and reduced linewidth are evidence of lateral confinement of excitons in monolayer WSe_2, demonstrating the versatility of our fabrication process.

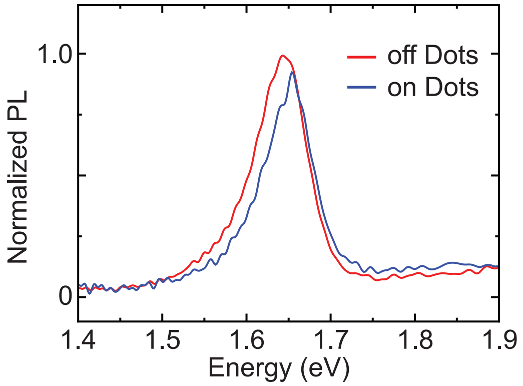

Figure S8: PL spectrum from a WSe_2 monolayer patterned with the same process as for the MoS_2 devices described in the manuscript.

PL spectrum of sparse nanodot sample

To further confirm that the linewidth of the nanodot PL is dominated by material inhomogeneity, we pattered a sample with sparse nanodots on monolayer WSe_2. We measured PL simultaneously from a few (2– 4) nanodots by confocal microscopy. As shown in Fig. S9, the PL from this sparse nanodot sample shows a similar broad linewidth similar as is measured from a larger ensemble as shown in the main text. This implies that the inhomogeneous linewidth is not due to the nanopatterning, but the inhomogeneous broadening of the monolayer material.

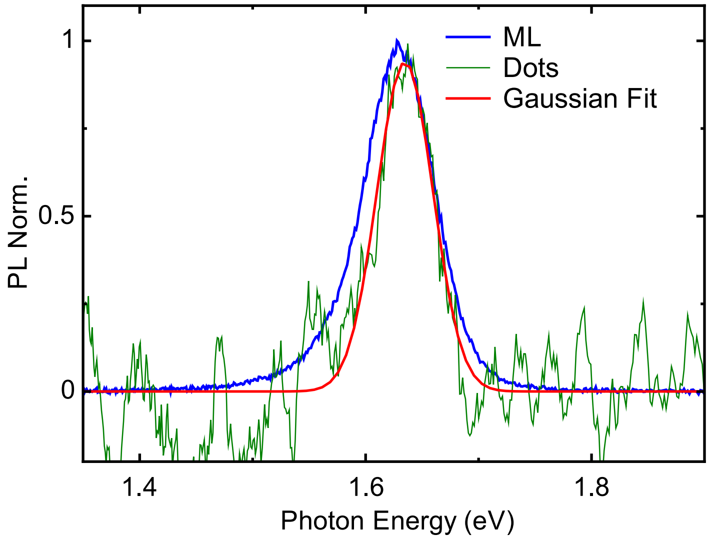

Figure S9: PL spectrum from a few sparse dots pattered on WSe_2 monolayer (green data with red fit for clarity) compared to the unpatterned monolayer (blue).

References

[1] Castellanos-Gomez, A., Buscema, M., Molenaar, R., Singh, V., Janssen, L., van der Zant, H. S. & Steele, G. A. Deterministic transfer of two-dimensional materials by all-dry viscoelastic stamping. *2D Mater.* **1**, 011002 (2014).

[2] Wei, G., Stanev, T. K., Czaplewski, D. A., Jung, I. W. & Stern, N. P. Silicon-nitride photonic circuits interfaced with monolayer MoS_2. *Appl. Phys. Lett.* **107**, 091112 (2015).

[3] Canet-Ferrer, J., Coronado, E., Forment-Aliaga, A. & Pinilla-Cienfuegos, E. Correction of the tip convolution effects in the imaging of nanostructures studied through scanning force microscopy. *Nanotechnology* **25**, 395703 (2014).

[4] Zhang, X., Qiao, X.-F., Shi, W., Wu, J.-B., Jiang, D.-S. & Tan, P.-H. Phonon and raman scattering of two-dimensional transition metal dichalcogenides from monolayer, multilayer to bulk material. *Chem. Soc. Rev.* **44**, 2757–2785 (2015).

[5] Saito, R., Tatsumi, Y., Huang, S., Ling, X. & Dresselhaus, M. Raman spectroscopy of transition metal dichalcogenides. *J. Physics: Cond. Mat.* **28**, 353002 (2016).

[6] Mignuzzi, S., Pollard, A. J., Bonini, N., Brennan, B., Gilmore, I. S., Pimenta, M. A., Richards, D. & Roy, D. Effect of disorder on raman scattering of single-layer MoS_2. *Phys. Rev. B* **91**, 195411 (2015).

[7] Conley, H. J., Wang, B., Ziegler, J. I., Haglund Jr, R. F., Pantelides, S. T. & Bolotin, K. I. Bandgap engineering of strained monolayer and bilayer MoS_2. *Nano Lett.* **13**, 3626–3630 (2013).

[8] Faraci, G., Gibilisco, S., Russo, P., Pennisi, A. R. & La Rosa, S. Modified raman confinement model for Si nanocrystals. *Phys. Rev. B* **73**, 033307 (2006).

[9] Faraci, G., Gibilisco, S. & Pennisi, A. R. Quantum confinement and thermal effects on the raman spectra of Si nanocrystals. *Phys. Rev. B* **80**, 193410 (2009).

[10] Swamy, V., Kuznetsov, A., Dubrovinsky, L. S., Caruso, R. A., Shchukin, D. G. & Muddle, B. C. Finite-size and pressure effects on the raman spectrum of nanocrystalline anatase TiO_2. *Phys. Rev. B* **71**, 184302 (2005).

[11] Ryu, S., Maultzsch, J., Han, M. Y., Kim, P. & Brus, L. E. Raman spectroscopy of lithographically patterned graphene nanoribbons. *ACS Nano* **5**, 4123–4130 (2011).

[12] Bischoff, D., Güttinger, J., Dröscher, S., Ihn, T., Ensslin, K. & Stampfer, C. Raman spectroscopy on etched graphene nanoribbons. *J. Appl. Phys.* **109**, 073710 (2011).

[13] You, Y., Ni, Z., Yu, T. & Shen, Z. Edge chirality determination of graphene by raman spectroscopy. *Appl. Phys. Lett.* **93**, 163112 (2008).

[14] Manoogian, A. & Woolley, J. Temperature dependence of the energy gap in semiconductors. *Can. J. Phys.* **62**, 285–287 (1984).

[15] Kaasbjerg, K., Thygesen, K. S. & Jacobsen, K. W. Phonon-limited mobility in n-type single-layer MoS_2 from first principles. *Phys. Rev. B* **85**, 115317 (2012).

[16] Zhao, H., Wachter, S. & Kalt, H. Effect of quantum confinement on exciton-phonon interactions. *Phys. Rev. B* **66**, 085337 (2002).

[17] Moody, G. *et al.* Intrinsic homogeneous linewidth and broadening mechanisms of excitons in monolayer transition metal dichalcogenides. *Nature Commun.* **6**, 8315 (2015).

[18] Mak, K. F., He, K., Shan, J. & Heinz, T. F. Control of valley polarization in monolayer MoS_2 by optical helicity. *Nature Nanotech.* **7**, 494–498 (2012).

[19] Lui, C., Frenzel, A., Pilon, D., Lee, Y.-H., Ling, X., Akselrod, G., Kong, J. & Gedik, N. Trion-induced negative photoconductivity in monolayer MoS_2. *Phys. Rev. Lett.* **113**, 166801 (2014).

[20] Zhang, C., Wang, H., Chan, W., Manolatou, C. & Rana, F. Absorption of light by excitons and trions in monolayers of metal dichalcogenide MoS_2: Experiments and theory. *Phys. Rev. B* **89**, 205436 (2014).

[21] Mak, K. F., He, K., Lee, C., Lee, G. H., Hone, J., Heinz, T. F. & Shan, J. Tightly bound trions in monolayer MoS_2. *Nature Mater.* **12**, 207–211 (2013).

[22] Suk, J. W., Lee, W. H., Lee, J., Chou, H., Piner, R. D., Hao, Y., Akinwande, D. & Ruoff, R. S. Enhancement of the electrical properties of graphene grown by chemical vapor deposition via controlling the effects of polymer residue. *Nano Lett.* **13**, 1462–1467 (2013).

[23] Huang, J., Hoang, T. B. & Mikkelsen, M. H. Probing the origin of excitonic states in monolayer WSe_2. *Sci. Rep.* **6** (2016).

[24] Cheng, R., Li, D., Zhou, H., Wang, C., Yin, A., Jiang, S., Liu, Y., Chen, Y., Huang, Y. & Duan, X. Electroluminescence and photocurrent generation from atomically sharp WSe_2/MoS_2 heterojunction p–n diodes. *Nano Lett.* **14**, 5590–5597 (2014).

[25] Zhou, H. *et al.* Large area growth and electrical properties of p-type WSe_2 atomic layers. *Nano Lett.* **15**, 709–713 (2014).

[26] Corfdir, P., Levrat, J., Dussaigne, A., Lefebvre, P., Teisseyre, H., Grzegory, I., Suski, T., Ganière, J.-D., Grandjean, N. & Deveaud-Plédran, B. Intrinsic dynamics of weakly and strongly confined excitons in nonpolar nitride-based heterostructures. *Phys. Rev. B* **83**, 245326 (2011).

[27] Cheiwchanchamnangij, T. & Lambrecht, W. R. L. Quasiparticle band structure calculation of monolayer, bilayer, and bulk MoS_2. *Phys. Rev. B* **85**, 205302 (2012).

[28] Peelaers, H. & Van de Walle, C. G. Effects of strain on band structure and effective masses in MoS_2. *Phys. Rev. B* **86**, 241401 (2012).

[29] Ramasubramaniam, A. Large excitonic effects in monolayers of molybdenum and tungsten dichalcogenides. *Phys. Rev. B* **86**, 115409 (2012).

[30] Ugeda, M. M. *et al.* Giant bandgap renormalization and excitonic effects in a monolayer transition metal dichalcogenide semiconductor. *Nature Mater.* **13**, 1091–1095 (2014).

[31] Lin, Y., Ling, X., Yu, L., Huang, S., Hsu, A. L., Lee, Y.-H., Kong, J., Dresselhaus, M. S. & Palacios, T. Dielectric screening of excitons and trions in single-layer mos_2. *Nano Lett.* **14**, 5569–5576 (2014). PMID: 25216267, `http://dx.doi.org/10.1021/nl501988y`.

[32] Robert, C. *et al.* Exciton radiative lifetime in transition metal dichalcogenide monolayers. *Phys. Rev. B* **93**, 205423 (2016).

[33] Bockelmann, U. & Bastard, G. Phonon scattering and energy relaxation in two-, one-, and zero-dimensional electron gases. *Phys. Rev. B* **42**, 8947–8951 (1990).

[34] Wagner, H. P., Schätz, A., Maier, R., Langbein, W. & Hvam, J. M. Interaction and dephasing of center-of-mass quantized excitons in wide znse/$zn_{0.94}mg_{0.06}Se$ quantum wells. *Phys. Rev. B* **57**, 1791–1796 (1998).

[35] Ip, A. H. *et al.* Hybrid passivated colloidal quantum dot solids. *Nature Nanotech.* **7**, 577–582 (2012).

[36] Chuang, C.-H. M., Brown, P. R., Bulovic, V. & Bawendi, M. G. Improved performance and stability in quantum dot solar cells through band alignment engineering. *Nature Mater.* **13**, 796–801 (2014).

[37] Ning, Z. *et al.* Air-stable n-type colloidal quantum dot solids. *Nature Mater.* **13**, 822–828 (2014).

[38] Sykora, M., Koposov, A. Y., McGuire, J. A., Schulze, R. K., Tretiak, O., Pietryga, J. M. & Klimov, V. I. Effect of air exposure on surface properties, electronic structure, and carrier relaxation in pbse nanocrystals. *ACS Nano* **4**, 2021–2034 (2010).

[39] Ihly, R., Tolentino, J., Liu, Y., Gibbs, M. & Law, M. The photothermal stability of PbS quantum dot solids. *ACS Nano* **5**, 8175–8186 (2011).
